# High-content image screening to identify chemical modulators for peroxisome and ferroptosis

**DOI:** 10.1186/s11658-024-00544-2

**Published:** 2024-02-17

**Authors:** Daheng Zheng, Fei Li, Shanshan Wang, Pu-Ste Liu, Xin Xie

**Affiliations:** 1 School of Life and Environmental Sciences, Shaoxing University, Shaoxing City, Zhejiang China; 2 School of Life Sciences and Biopharmaceutics, Guangdong Pharmaceutical University, Guangdong, China; 3 Department of Biochemistry and Molecular Biology, College of Medicine, National Cheng Kung University, Tainan, Taiwan, ROC

**Keywords:** Peroxisome, Homeostasis, U-2OS, High-content screening, Ferroptosis, Oxidative stress, Target selective inhibitor

## Abstract

**Background:**

The peroxisome is a dynamic organelle with variety in number, size, shape, and activity in different cell types and physiological states. Recent studies have implicated peroxisomal homeostasis in ferroptosis susceptibility. Here, we developed a U-2OS cell line with a fluorescent peroxisomal tag and screened a target-selective chemical library through high-content imaging analysis.

**Methods:**

U-2OS cells stably expressing the mOrange2-Peroxisomes2 tag were generated to screen a target-selective inhibitor library. The nuclear DNA was counterstained with Hoechst 33342 for cell cycle analysis. Cellular images were recorded and quantitatively analyzed through a high-content imaging platform. The effect of selected compounds on ferroptosis induction was analyzed in combination with ferroptosis inducers (RSL3 and erastin). Flow cytometry analysis was conducted to assess the level of reactive oxygen species (ROS) and cell death events.

**Results:**

Through the quantification of DNA content and peroxisomal signals in single cells, we demonstrated that peroxisomal abundance was closely linked with cell cycle progression and that peroxisomal biogenesis mainly occurred in the G1/S phase. We further identified compounds that positively and negatively regulated peroxisomal abundance without significantly affecting the cell cycle distribution. Some compounds promoted peroxisomal signals by inducing oxidative stress, while others regulated peroxisomal abundance independent of redox status. Importantly, compounds with peroxisome-enhancing activity potentiated ferroptosis induction.

**Conclusions:**

Our findings pinpoint novel cellular targets that might be involved in peroxisome homeostasis and indicate that compounds promoting peroxisomal abundance could be jointly applied with ferroptosis inducers to potentiate anticancer effect.

**Graphical Abstract:**

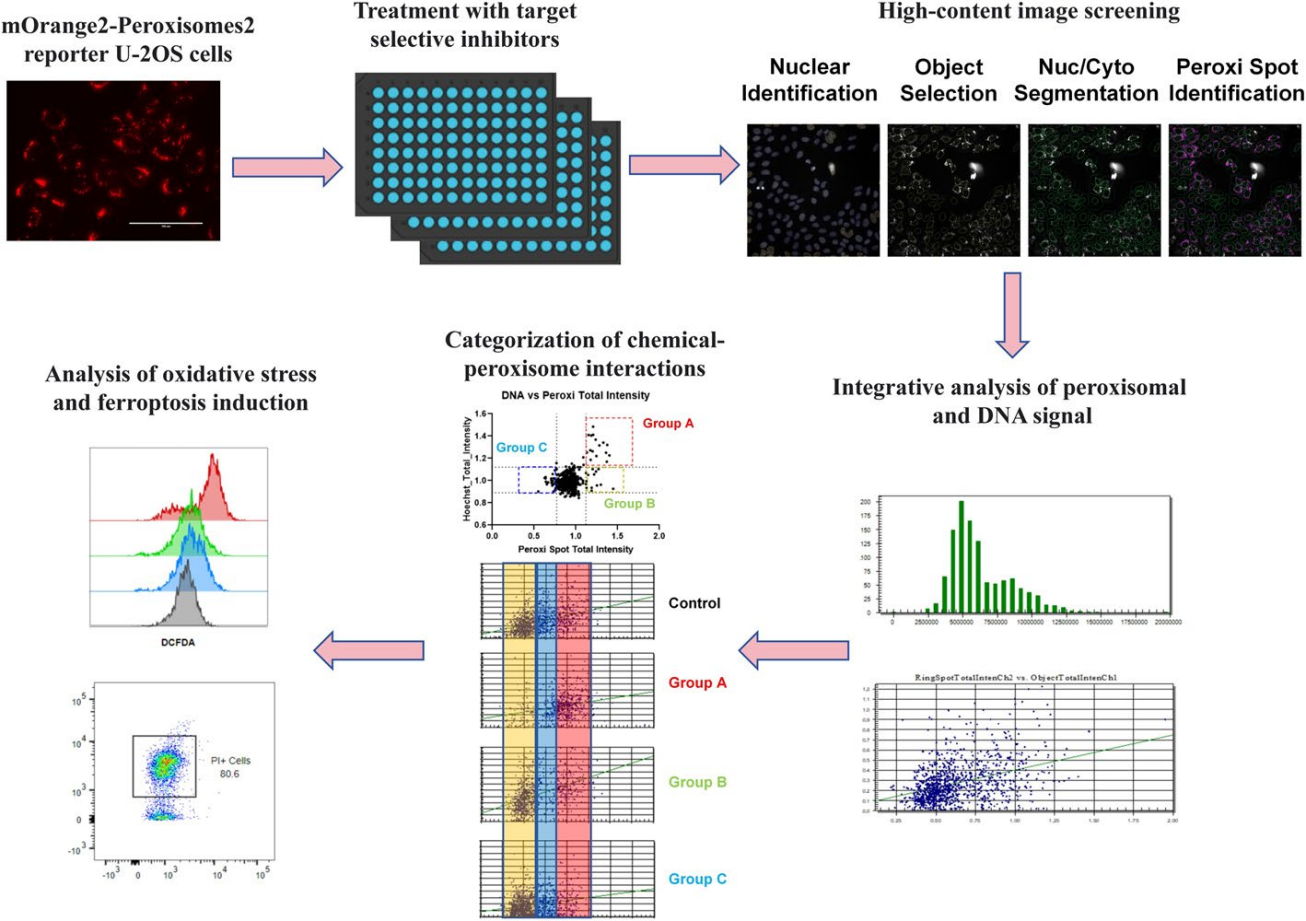

## Introduction

In eukaryotic cells, the peroxisome is a conserved organelle implicated in cellular metabolism, redox regulation, and the development of human metabolic diseases [1]. Peroxisomes are important sites for branched chain fatty acid β-oxidation, ether glycerolipid biosynthesis, and hydrogen peroxide metabolism [2, 3]. In addition, peroxisomes interact with other organelles to regulate a variety of cellular processes; For instance, extensive communication between peroxisomes and mitochondria coordinates the integrated lipid metabolism [4, 5]. Peroxisome-anchored mitochondrial antiviral signaling protein (MAVS) cooperates with mitochondrial signaling in mounting cellular defense against viral infection [6].

Peroxisomes are highly dynamic organelles with a high degree of variety in number, size, shape, and protein composition in different cell types and physiological states. In response to cellular stress, peroxisomal gene expression is upregulated and the shape shows elongation [7–9]. In cancer cells or cells with elevated lipid catabolism, the number of peroxisomes is increased to meet the increased energy demands [2, 10]. A recent study has demonstrated that the increase of peroxisomal abundance induces the stem cell differentiation to promote intestinal epithelial repair, opening up new treatment avenues for patients with inflammatory bowel disease [11]. Besides, peroxisomal β-oxidation serves as an intracellular fatty acid sensor to regulate lipolysis [12]. Peroxisomal dysfunction is also implicated in different pathophysiological conditions, including insulin production from pancreatic beta-cells, nonalcoholic fatty liver disease, neurodegeneration, and cancer [12–15].

The generation of peroxisomes in mammalian cells mainly occurs through two parallel pathways: de novo biogenesis and asymmetric division [1]. De novo synthesis refers to the fusion of mitochondria-derived vesicles and endoplasmic reticulum-derived pre-peroxisomes [16]. Damaged or redundant peroxisomes are eliminated through the autophagy pathway (pexophagy) [17]. The biosynthesis and degradation of peroxisomes are highly coordinated to maintain organelle homeostasis. For example, excessive peroxisomal metabolites can serve as signals to inhibit peroxisomal generation and promote pexophagy [18]. Currently, more than 30 peroxins (PEXs) and peroxisomal membrane proteins have been characterized in the functional homeostasis of peroxisomes, including membrane protein assembly, matrix protein import, metabolite transport, lipid metabolism, organelle division, and pexophagy [1, 19, 20]. Defects in 14 genes encoding peroxisomal membrane proteins, matrix protein import, and division have been found to cause peroxisome biogenesis disorders (PBDs), such as Zellweger disorder, Heimler syndrome, and rhizomelic chondrodysplasia punctata (RCDP) [21]. In addition, peroxisome-dependent synthesis of ether-linked phospholipids affects the sensitivity of cancer cells to ferroptosis, which can be employed as an anticancer strategy [22]. Nevertheless, how cells integrate different signals to regulate peroxisomal homeostasis remains largely unknown.

In this study, we established a peroxisome reporter cell line to screen a target-selective inhibitor library through high-content imaging profiling. We identified the candidates that were able to positively or negatively regulate peroxisomal abundance, and validated the compounds with pro-ferroptosis potential.

## Methods

### Cell culture and treatment

Human osteosarcoma cell line U-2OS (catalog no. CL-0236) and human cervical cancer cell line HeLa (catalog no. CL-0101) were obtained from Procell Life Science & Technology Co., Ltd. (Wuhan, China). The cell lines were authenticated by the supplier through STR profiling. The cells were cultivated in McCoy’s 5A medium (PM150710; Procell) supplemented with 10% fetal bovine serum (PM164210; Procell) and 1 × penicillin–streptomycin solution (PB180120; Procell) at 37 °C and 5% CO_2_. To generate peroxisome-reporter cell line, 5 × 10^5^ parental U-2OS cells were inoculated into a six-well plate and transfected with 4 µg of mOrange2-Peroxisomes2 vector (54596; Addgene, MA, USA) using Lipofectamine 2000 reagent (11668019, Invitrogen, Shanghai, China). At 48 h after the transfection, cells were selected in the medium containing 400 μg/ml G418 (HY-K1056; MedChemExpress, Shanghai, China) for 2–3 weeks to generate cell clones stably expressing the Orange2-Peroxisome tag. RSL3 (HY-100218A; MedChemExpress) and erastin (HY-15763; MedChemExpress) were used as ferroptosis inducers at 1 μM and 2 μM for 24 h treatment, respectively. Ferrostatin-1 (Fer-1, HY-100579; MedChemExpress) was applied at 2 μM to inhibit ferroptosis.

### High-content screening

U-2OS cells stably expressing the Orange2-Peroxisome fluorescent tag were used for high-content screening analysis within ten passages. The cells were cultured in 96-well black/clear bottom assay plates (137101; ThermoFisher Scientific, CA, USA) at a density of 5000 cells/well. Cells were treated with 1 μM of chemicals from a target-selective inhibitor library (L3500; Selleck Chemicals LLC, TX, USA) that contains about 600 highly selective inhibitors covering over 123 protein targets. An equal volume of dimethylsulfoxide (DMSO) was applied as the control, and three biological replicates were prepared for the screening. Cells were treated for 24 h and then fixed in 3.7% formaldehyde for 10 min at ambient temperature. Afterward, the fixed cells were rinsed with PBS and counterstained with Hoechst 33342 (10 μM, HY-15559; MedChemExpress) for 15 min at ambient temperature. Stained cells were scanned using the EVOS M7000 automated imaging platform (ThermoFisher Scientific), with a × 20 objective (Olympus™ 20X Objective, X-Apo, 0.80NA/0.6WD) and an light-emitting diode (LED) light source for sequential fluorescence imaging. The Hoechst signal was captured in the DAPI channel, and the mOrange2-Peroxisome signal was recorded in the RFP channel. A total of 12 fields in each well were captured, and Compartment Analysis Bio Application in the Cellomics HCS Studio software (ThermoFisher Scientific) was adopted for quantitative image analysis. Hoechst-stained nuclei were identified as primary objects, and a simulated cytoplasm was created on the basis of nuclear shape and neighboring cells. Peroxisomal signals in the cytoplasmic region were integrated as the total peroxisomal signal in each cell. The integrated Hoechst staining intensity was used as the indicator of cell cycle distribution.

### Immunofluorescence (IF) staining

As an intrinsic peroxisomal marker for IF staining, 70-kDa peroxisomal membrane protein (PMP70) was used. U-2OS and HeLa cells were cultured in 96-well black/clear bottom assay plate at a density of 5000 cells/well. Cells were treated with 1 μM of chemicals or DMSO for 24 h and fixed in 3.7% formaldehyde for 10 min at ambient temperature. The cells were then permeabilized with 0.025% Triton X100 for 15 min, followed by blocking with 2.5% bovine serum albumin (HY-D0842; MedChemExpress). Anti-PMP70 antibody (ab85550; Abcam, Cambridge, UK) was diluted at 1:1000 in PBS and applied in the permeabilized cells for 18 h at 4 °C. After rinsing with PBS, the cells were further labeled with anti-rabbit IgG H&L (Alexa Fluor^®^ 488 conjugated) (ab150077; 1:3000, Abcam) for 1 h at ambient temperature. The cells were counterstained with Hoechst 33342 (10 μM, HY-15559, MedChemExpress) for 15 min. The images were captured on the EVOS M7000 automated imaging platform and analyzed using the Compartment Analysis Bio Application in the Cellomics HCS Studio software (Thermo Fisher Scientific).

### Western blot

U-2OS and HeLa cells cultured at 70% confluence in a six-well plate were treated with 1 μM of chemicals or DMSO for 24 h. Protein samples were extracted from cultured cells using radioimmunoprecipitation assay buffer (RIPA) lysis buffer (P0013K; Beyotime, Beijing, China) at 4 °C for 15 min, and the protein concentration was quantified using a bicinchoninic acid protein assay kit (P0012; Beyotime). In 10% polyacrylamide gels, 10 μg of protein sample was separated and transferred to the polyvinylidene fluoride membrane. After being blocked with 5% nonfat milk for 2 h, the membrane was incubated with primary antibodies (anti-PEX3, ab74505, 1:1000; and anti-β-actin, ab8227, 1:2000; Abcam, Cambridge, UK) overnight at 4 °C. The membrane was further probed with the HRP-conjugated secondary antibody (ab288151, 1: 4000) for 1 h. Protein bands were developed using the BeyoECL Plus chemiluminescence kit (P0018M; Beyotime).

### Flow cytometry analysis

U-2OS cells with different treatment were trypsinized and resuspended in fresh medium containing propidium iodide (PI) (10 μg/ml, ST511; Beyotime) for cell death quantification, and PI+ cells were detected after 10 min staining. For reactive oxygen species (ROS) measurement, cells were labeled with 2′,7′-dichlorodihydrofluorescein diacetate (DCFDA) (1 μM, C6827; Thermo Fisher Scientific) for 45 min. To determine the response to oxidative stress, cells were pulsed with 200 μM H_2_O_2_ for 30 min before DCFDA staining. Cell cycle distribution was analyzed in live cells after staining with Hoechst 33342 (10 μM) for 20 min. Cell event analysis was conducted using a BD FACSAria III flow cytometer (BD Biosciences, CA, USA).

### Three-dimensional spheroid culture

The 3D culture of tumor spheroid was established by seeding U-2OS cells at a density of 10^3^ cells/well in a U-bottom 96-well plate coated with Matrigel (356237; Corning, CA, USA). The cells were cultured at 37 °C and 5% CO_2_ for 3–4 days until the emergence of individual spheroids. Spheroids were treated with RSL3 (1 μM) or in combination with 1 μM MLN2238 from the chemical library in complete culture medium for 24 h. After treatment, the spheroids were stained with Hoechst 33342 (10 μM) and PI (10 μg/ml) for 10 min and imaged under the EVOS M7000 platform.

### Statistics

All the results are summarized as mean ± standard deviation. Unpaired Student’s *t* test was employed to compare two conditions. Multiple comparisons were performed by one-way analysis of variance with Tukey’s post hoc test. The difference was considered to be statistically significant when *p* < 0.05.

## Results

### Screening of the target-selective chemical library using the peroxisome-labeled U-2OS cell line

**Fig. 1:**
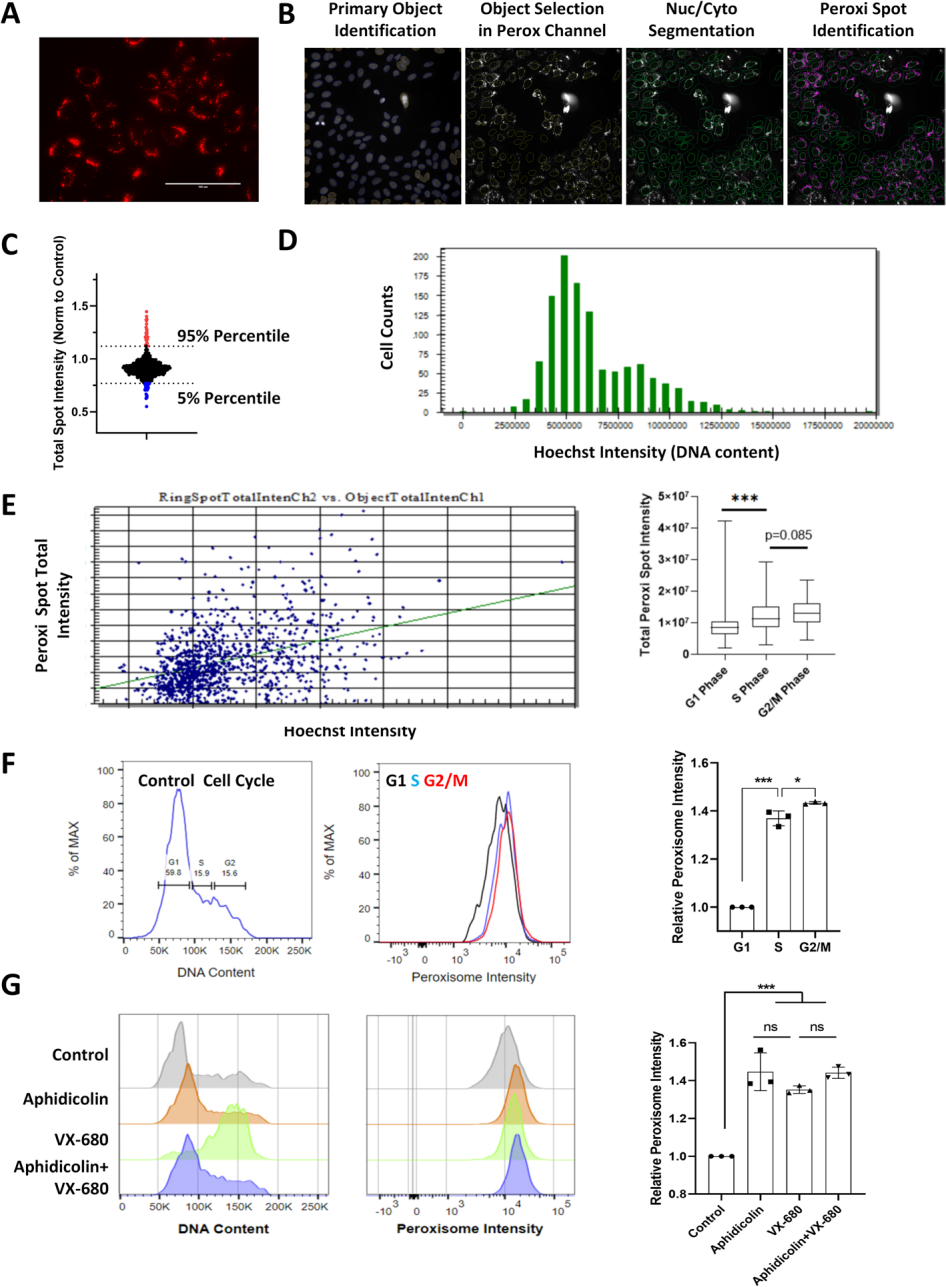
High-content image analysis of cell-cycle-dependent peroxisomal signal in U-2OS cells expressing the mOrange2-Peroxisomes2 marker. **A** Representative image of U-2OS cell line with mOrange2-Peroxisomes2 fluorescent label, scale bar: 100 μm. **B** High-content image analysis workflow of single-cell detection and peroxisomal signal quantification. Single cell nuclei were detected based on the Hoechst staining (blue markers), while clustered nuclei or the nuclei at the edge of images (yellow markers) were excluded from analysis (primary object identification). Afterward, selected single cells were segmented into cytoplasmic and nuclear regions (nuc/cyto segmentation), and the peroxisomal signal in the cytoplasm was quantified (peroxi spot identification). **C** The peroxisomal signals after the treatment with compounds (1 μM, 24 h) from the target-selective chemical library were normalized to the control average (DMSO treatment). Blue: compounds reducing peroxisomal signals. Red: compounds increasing peroxisomal signals. **D** Histogram showing the distribution of Hoechst DNA staining signals in the control cell population. **E** Peroxisomal spot total intensity was plotted against the Hoechst signal in individual cells of the control sample. The distribution of peroxisomal spot total intensity per cell was summarized in cell populations of different cell cycle phases. **F** Flow cytometry analysis of U-2OS cells with peroxisomal fluorescent marker and Hoechst staining. The relative levels of peroxisomal intensities in each cell cycle phases were normalized against G1 cell population. **G** U-2OS cells were treated with aphidicolin (DNA synthesis inhibitor, 1 μM), VX-680 (Aurora Kinase inhibitor, 1 μM) or aphidicolin plus VX-680 for 24 h. The cell cycle distribution and peroxisomal signals were analyzed by flow cytometry. The relative levels of peroxisomal intensities in each sample were normalized against the control cell population. *n* = 3 independent experiments. *p < 0.05; ***p < 0.001

We initially established a U-2OS cell line with genetically labeled peroxisome using the mOrange2-Peroxisomes-2 vector (mOrange2 fused with peroxisome targeting signal 1) [23]. The fluorescent tag of peroxisome was clearly visible in the cytoplasm (Fig. 1A). Fixed cells were imaged using an EVOS M7000 automated microscope. Cellomics software was adopted to perform single-cell nucleocytoplasmic compartmentalization and quantitative fluorescence analysis on the images. Single-cell nuclei were located based on the Hoechst staining (blue markers), while clustered nuclei or the nuclei at the edge of images (yellow markers) were excluded from analysis (Fig. 1B, primary object identification). Single cells were segmented into cytoplasmic and nuclear regions (Fig. 1B, nuc/cyto segmentation), and finally peroxisomal abundance in the cytoplasm was quantified by integrating the fluorescent spot signals (Fig. 1B, peroxi spot identification). We screened a target selective inhibitor library containing about 600 compounds (1 μM treatment for 24 h, three biological replicates), with DMSO as the negative control. The chemicals that induced strong cytotoxicity (cell counts less than 25% of control average: there were about 2000 cells imaged in each control sample) were removed, since the limited number of cells might not provide enough data for quantification. Peroxisomal signal in each treatment was normalized against the control average, and we identified chemicals that increased or reduced peroxisomal signals in U-2OS cells (Fig. 1C).

### Cell-cycle-dependent peroxisomal biogenesis

The distribution of Hoechst staining intensity in single cell population manifested the cell cycle distribution (Fig. 1D). The analysis of Hoechst staining and peroxisomal signals showed a positive correlation, with elevated level of peroxisomal signals as the DNA content increased (Fig. 1E). We also performed flow cytometry analysis in live U-2OS cells with peroxisomal marker and Hoechst staining. There was also an increasing trend of peroxisomal intensity as the cell cycle progressed (Fig. 1F). Of note, a major increase of peroxisomal signal was observed between the G1 and S phase transition (Fig. 1F). To confirm that G1 is the major cell cycle phase for peroxisomal biogenesis, U-2OS cells were treated with aphidicolin (DNA synthesis inhibitor, 1 μM), VX-680 (Aurora kinase inhibitor, 1 μM) or aphidicolin plus VX-680 for 24 h. Aphidicolin or aphidicolin plus VX-680 arrested the major cell population at the G1/S transition, and VX-68 induced G2/M arrest (Fig. 1G). Cell cycle arrest at the G1/S phase significantly increased the peroxisomal signals. However, cells blocked in the G2/M phase did not show further increase of peroxisomal signals (Fig. 1G). Therefore, the biogenesis of peroxisomes occurs mainly during the G1 to S phase transition. These findings also raise a concern regarding the impact of chemical-induced cell cycle change on peroxisomal abundance analysis.

### Categorization of chemical–peroxisome interactions

**Fig. 2:**
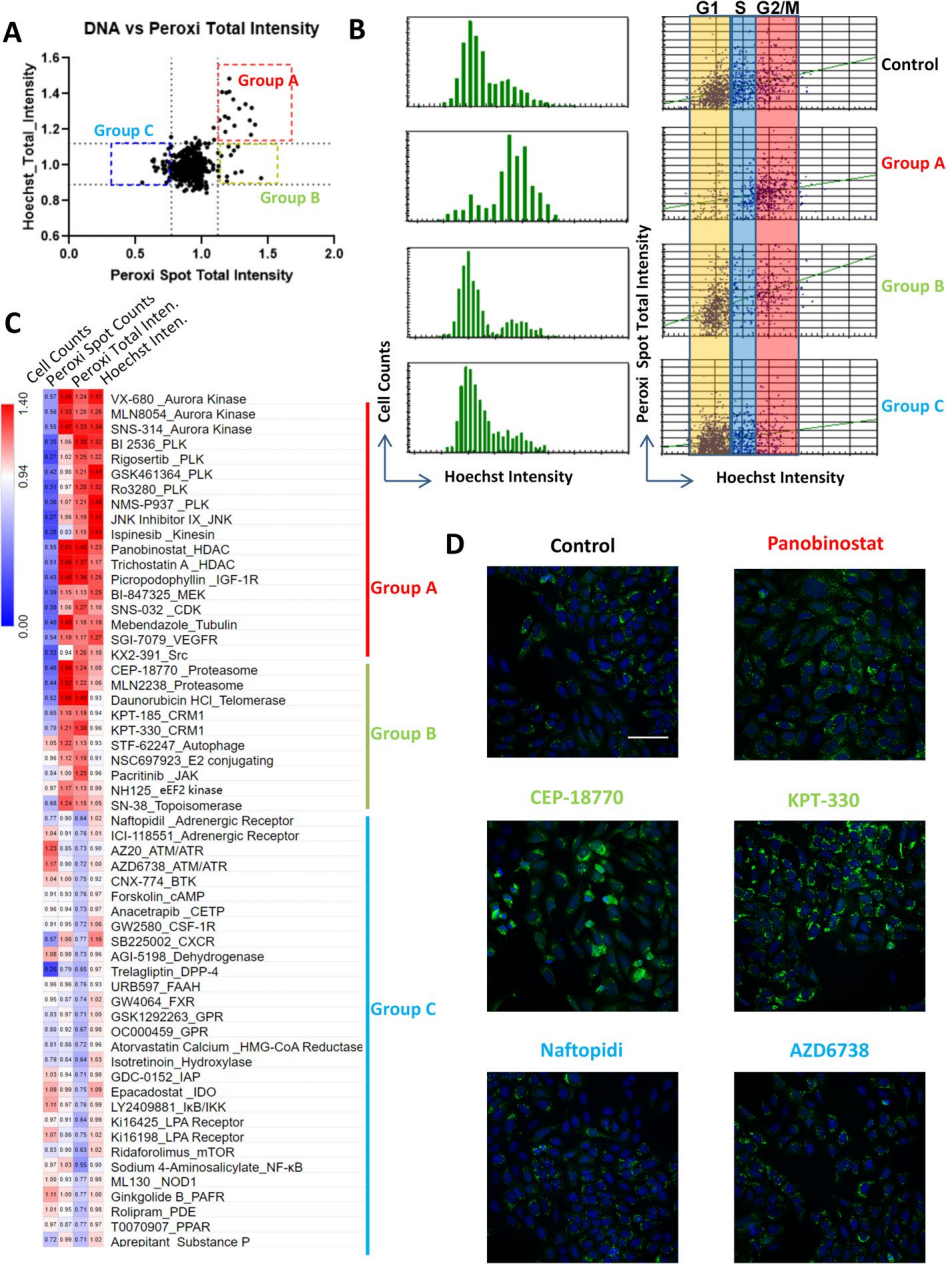
High-content screening results of a target-selective library in U-2OS cells expressing the mOrange2-Peroxisomes2 marker. **A** The average peroxisomal signal was plotted against Hoechst staining intensity for each chemical treatment. The signal of each channel was standardized by the mean value of the control sample. Data are summarized from three biological replicates. Group A: compounds that affect peroxisomes by altering cell cycle. Group B: compounds that increase peroxisomal abundance without altering cell cycle. Group C: compounds that reduce peroxisomal abundance without altering cell cycle. **B** Representative results of Hoechst intensity distribution and the relative peroxisomal signals in control and compounds from each group. **C** Heatmap showing the relative values (normalized to controls) of cell counts, peroxisomal spot counts per cell, peroxisomal total intensity per cell, and Hoechst intensity per cell of compound treatment in group A–C. **D** Representative images of control and chemical treatment from each group: Panobinostat: group A; CEP-18770 and KPT-330: group B; Naftopidi and AZD6738: group C. Scale bar: 100 μm

To exclude the impact of cell cycle change on peroxisomes, we plotted the peroxisomal signal against Hoechst staining intensity for each chemical treatment (the signal intensity was standardized by the mean values of control samples). We took the 95% and 5% percentile as the cutoff to distinguish the changes in DNA content (cell cycle) and peroxisomal abundance (Fig. 2A). Through the above analysis, the compounds could be categorized into three groups: group A, compounds that affect peroxisomes by altering cell cycle; group B, compounds that increase peroxisomal abundance without altering cell cycle; and group C, compounds that reduce peroxisomal abundance without altering cell cycle (Fig. 2A). Through a detailed comparison of the cell cycle distribution and peroxisomal intensity, we found that compounds in group A induced a peroxisomal increase by arresting the cell cycle in G2/M phase. Group B and C compounds did not significantly change cell cycle distribution. Group B compounds tended to increase peroxisomal signals in all cell cycle phases, while group C compounds repressed peroxisomal signals in all cell cycle phases (Fig. 2B).

The relative values (normalized to controls) of cell counts, peroxisomal spot counts per cell, peroxisomal total intensity per cell, and Hoechst intensity per cell are summarized in Fig. 2C. Representative images of each group are shown in Fig. 2D. Group A compounds are inhibitors of proteins involved in cell cycle progression and cell division, such as Aurora kinase, Polo-like kinase (PLK), c-Jun N-terminal kinase (JNK), kinesin, tubulin, mitogen-activated protein kinase (MEK), cyclin-dependent kinase (CDK), and histone deacetylase (HDAC). These chemicals reduced the cell counts but increased the total peroxisomal intensity and the total Hoechst staining signal, since these compounds arrested cell cycle progression. Some inhibitors increased peroxisomal spot counts per cell (such as aurora kinase inhibitors), while others (such as PLK inhibitors) did not altered the spot counts, indicating that different inhibitors may also affect the dynamic fission or fusion of peroxisomes.

Group B compounds promoted the total peroxisomal intensity without the increase of DNA content (Fig. 2C). Most of these compounds also increased the peroxisomal spot counts per cell. These chemicals contain inhibitors for proteasome, exportin 1 (CRM1), autophagy, ubiquitin-conjugating enzyme (E2) complex Ubc13-Uev1A, Janus kinase (JAK), and eukaryotic elongation factor 2 (eEF2) kinase.

Group C compounds attenuated the total peroxisomal intensity without significantly affecting the Hoechst staining intensity (Fig. 2C). Most of the compounds did not show strong effect on cell counts, except for ataxia-telangiesctasia mutated (ATM)/ataxia telangiectasia and Rad3 related (ATR) inhibitors, which increased cell counts, and dipeptidyl peptidase 4 (DPP-4) and CXC motif chemokine receptor 2 (CXCR2) inhibitor, which dramatically reduced cell counts. This group of compounds encompasses diverse targets, including ATM/ATR (DNA damage response), adrenergic receptor, cAMP synthesis, G-protein coupled receptor (GPR), lysophosphatidic acid (LPA) receptor, nuclear factor-kappa B (NF-kappa B) signaling, and different enzymes.

**Fig. 3:**
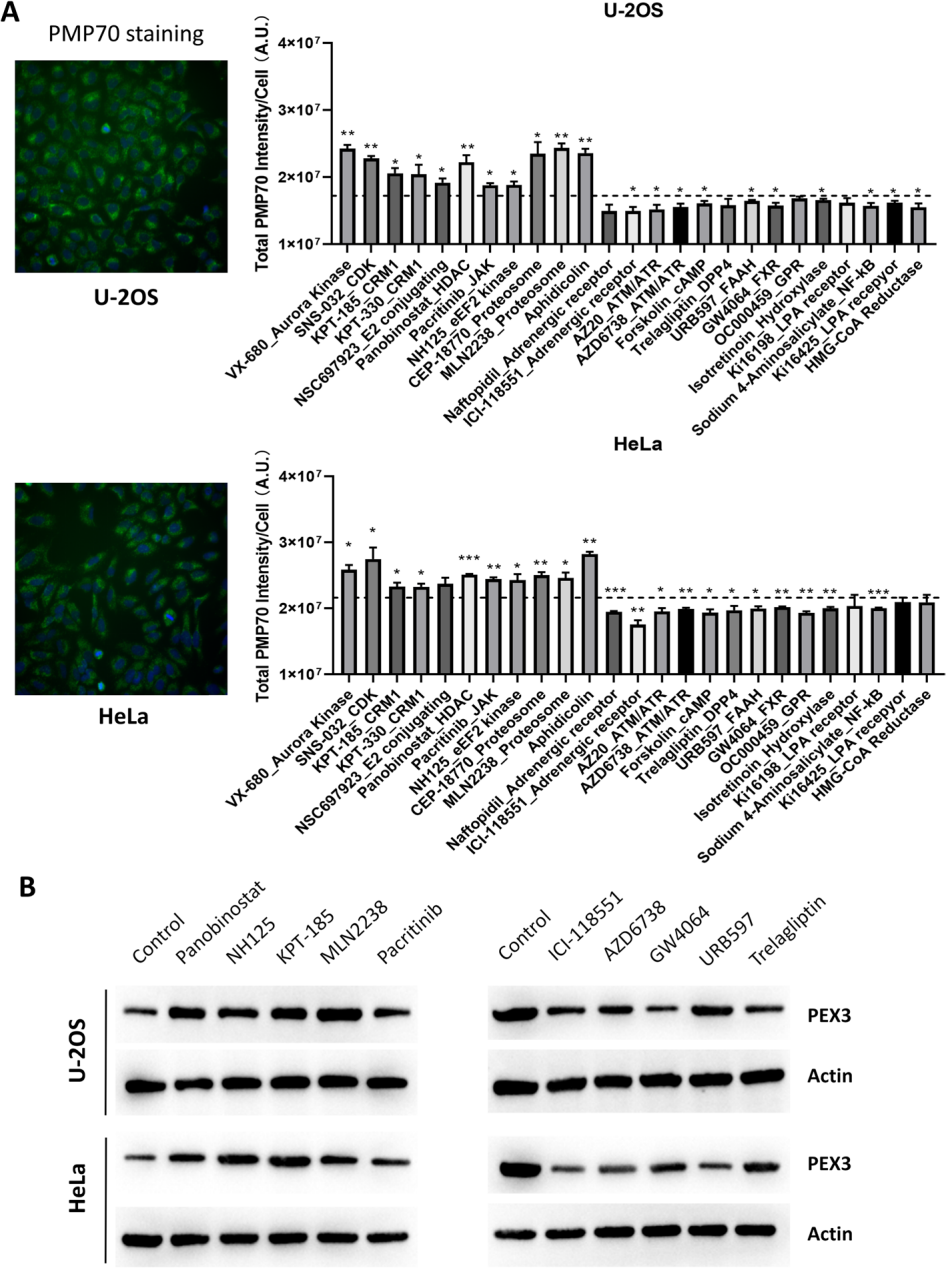
Validation of screening results by immunofluorescence (IF) staining and Western blot. **A** IF staining of intrinsic peroxisomal marker PMP70 in HeLa and U-2OS cell lines. High-content imaging analysis was applied to quantify PMP70 staining intensity after the treatment of selective compounds (1 μM, 24 h). Dotted line represents the mean value of control samples (DMSO treatment). Data are summarized from three biological replicates. *p < 0.05; **p < 0.01; ***p < 0.001. **B** Western blot analysis of PEX3 protein levels after the treatment of indicated compounds (1 μM, 24 h) in HeLa and U-2OS cell lines

Since the initial screening was conducted in genetically labeled U-2OS cell line, we further confirmed the effects of compounds from each group by immunofluorescence (IF) staining of the intrinsic peroxisomal marker PMP70 in HeLa and U-2OS cell lines. PMP70 is one of the major components of peroxisomal membranes, which belongs to the ATP binding cassette transporter superfamily. PMP70 is required for peroxisomal proliferation and fatty acid beta-oxidation [24]. High-content imaging quantification of IF staining showed results consistent with the screening results using genetically labeled U-2OS cell line (Fig. 3A). Inhibitors of cell cycle regulators (VX-680, SNS-032, aphidicolin) significantly increased the PMP70 staining intensity, as well as the inhibitors of CRM1, E2-conjugating enzyme, JAK, eEF2 kinase, and proteasome. Further, most of the inhibitors suppressing peroxisomal signals in the initial screening also reduced the PMP70 staining intensity in both HeLa and U-2OS cells (Fig. 3A). Besides, Western blot analysis also showed that chemicals promoting peroxisomal signal in high-content screening increased peroxin 3 (PEX3) expressions, while the ones reducing peroxisomal signal repressed PEX3 expressions (Fig. 3B). Therefore, IF staining of PMP70 and Western blot analysis of PEX3 further verified the impacts of different inhibitors on peroxisomes.

### Peroxisomal biogenesis and oxidative stress

**Fig. 4:**
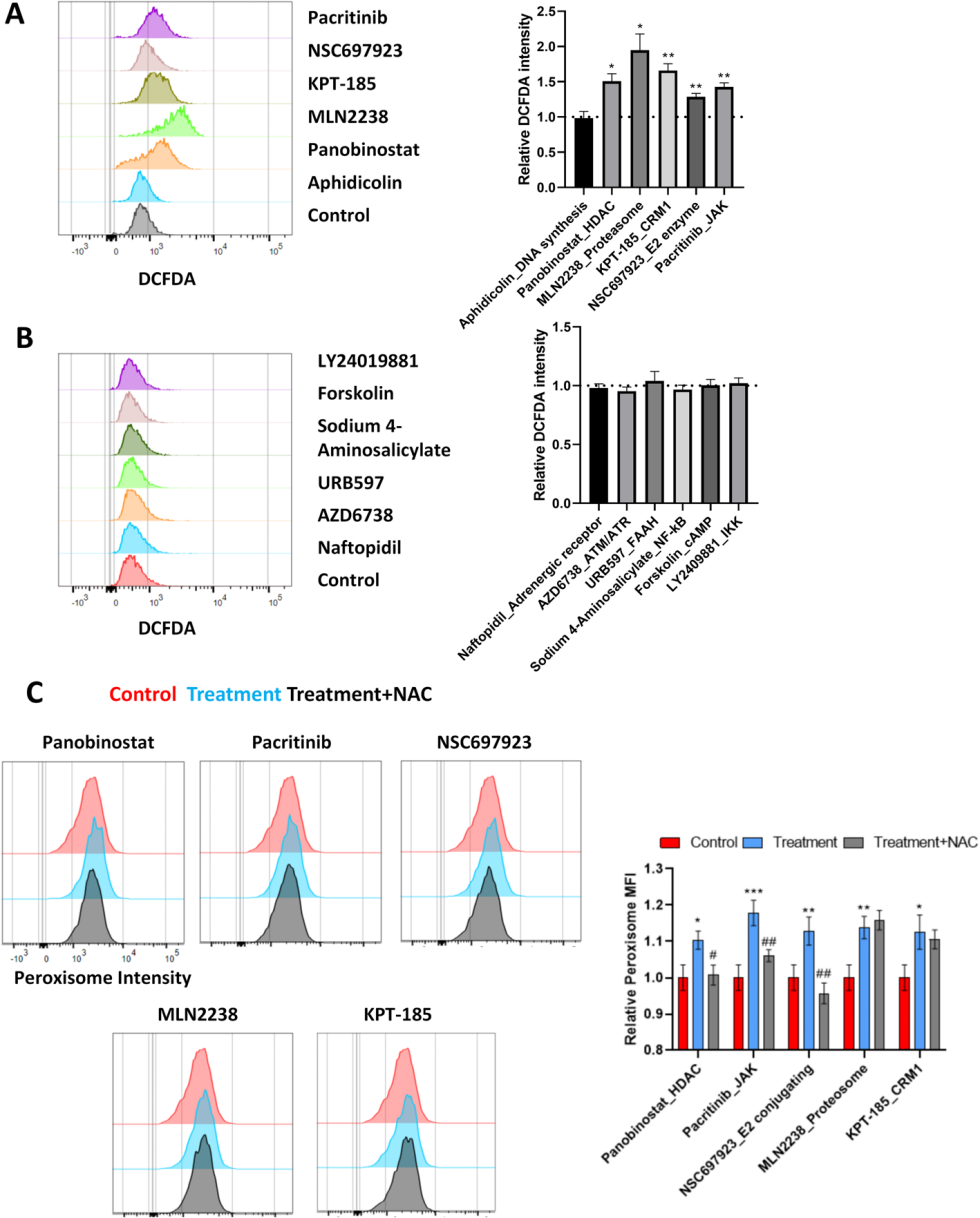
Peroxisomal abundance and oxidative stress. **A** Flow cytometry analysis of DCFDA staining (ROS sensor) in U-2OS cells after treatment with compounds that promoted peroxisomal abundance. **B** Flow cytometry analysis of DCFDA staining (ROS sensor) in U-2OS cells after treatment with compounds that reduced peroxisomal abundance. **C** U-2OS cells with peroxisomal fluorescent tag were treated with 1 μM panobinostat (HDAC inhibitor), pacritinib (JAK inhibitor), and NSC697923 (E2 conjugating enzyme inhibitor), MLN2238 (proteasome inhibitor) and KPT-185 (CRM1 inhibitor) in presence or absence of 2 mM NAC. The relative levels of peroxisomal signals were analyzed by flow cytometry. *N* = 3 independent experiments. *p < 0.05 versus control; **p < 0.01 versus control; ***p < 0.001 versus control; ^#^*p* < 0.05 versus treatment; ^##^*p* < 0.01 versus treatment

There is evidence that oxidative stress can trigger peroxisome biogenesis by upregulating PEX genes [8]. We therefore wondered whether these chemicals positively or negatively regulate peroxisomal abundance by impinging on the redox status. To this end, U-2OS cells with or without chemical treatment were subjected to DCFDA staining (ROS sensor) and flow cytometry analysis. The inhibitors that increased peroxisomal signals elevated ROS levels in U-2OS cells, except for aphidicolin (Fig. 4A). In contrast, chemicals that reduced peroxisomal signals did not significantly affect ROS levels (Fig. 4B). To investigate whether ROS neutralization could abolish the effect of chemicals that positively regulate peroxisomal signals, U-2OS cells with peroxisomal fluorescent tag were treated with each chemical in the presence of antioxidant NAC (*N*-acetyl cysteine). NAC significantly attenuated peroxisomal signals induced by panobinostat (HDAC inhibitor), pacritinib (JAK inhibitor), and NSC697923 (E2 conjugating enzyme inhibitor), while the effects of MLN2238 (proteasome inhibitor) and KPT-185 (CRM1 inhibitor) were not affected by NAC (Fig. 4C). These findings suggest that chemicals may promote peroxisomal abundance by incurring oxidative stress, while the ones that reduce peroxisomal abundance exert their effects in a ROS-independent manner. Proteasome and CRM1 inhibitors could promote peroxisomal abundance and increase ROS level; however, their effects on peroxisomes are not the consequence of oxidative stress.

### Peroxisome targeting compounds sensitize U-2OS cells to ferroptosis induction

**Fig. 5:**
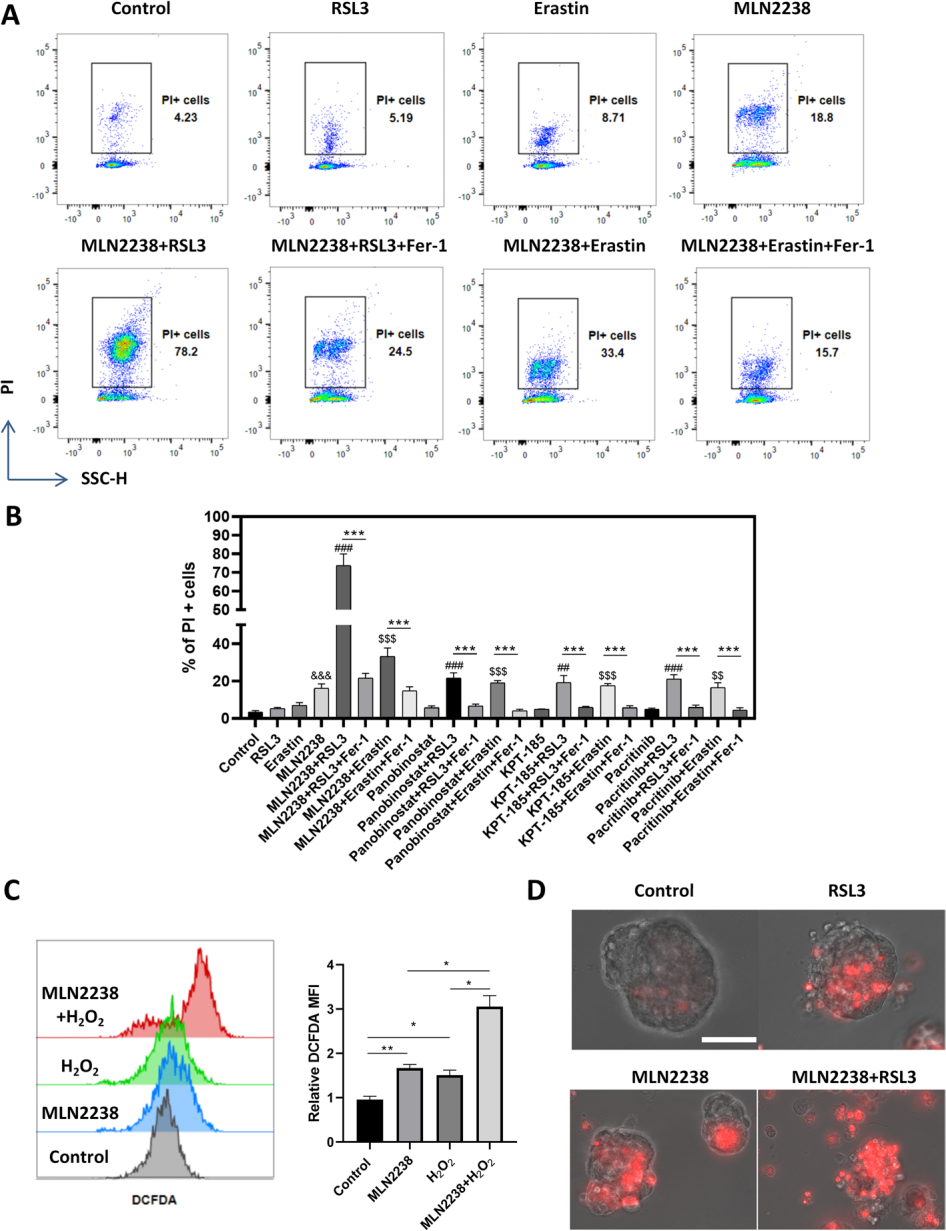
Peroxisome-promoting compounds sensitize U-2OS cells to ferroptosis induction. **A** U-2OS cells were treated with ferroptosis inducer (RSL3 (1 μM) or erastin (2 μM)), proteasome inhibitor MLN2238 (1 μM) or drug combination with/without ferroptosis inhibitor Fer-1 (2 μM) for 24 h. Cell death events were analyzed by PI staining. **B** Summary of percentage of cell death events in U-2OS cells after treatment with ferroptosis inducer (1 μM RSL3 or 2 μM erastin), chemicals (1 μM) or drug combination with/without ferroptosis inhibitor Fer-1 (2 μM). *N* = 3 independent experiments. ***p < 0.001 versus the Fer-1 treatment group; ^##^*p* < 0.01 and ^###^*p* < 0.001 versus RSL3 treatment; ^$$^*p* < 0.01 and ^$$$^*p* < 0.001 versus erastin treatment; ^&&&^*p* < 0.001 versus control group. **C** U-2OS cells with or without MLN2238 treatment (1 μM, 24 h) were exposed to 200 μM H_2_O_2_ for 30 min. Cells were labeled with DCFDA, and the relative ROS levels were determined by flow cytometry. *N* = 3 independent experiments. *p < 0.05; **p < 0.01. **D** Spheroids derived from U-2OS cells were treated with RSL3 (1 μM), MLN2238 (1 μM) or RSL3 + MLN2238 for 24 h. The spheroids were then stained with PI and imaged under EVOS 7000 microscope, scale bar: 100 μm

Since peroxisome-dependent biosynthesis of ether-linked phospholipids is essential for ferroptosis induction and silencing peroxisomal genes could confer resistance to ferroptosis [22, 25], we hypothesized that the chemicals promoting peroxisomal abundance could enhance the sensitivity to ferroptosis induction. We then treated U-2OS cells with ferroptosis inducers (RSL3 and erastin) alone or in combination with panobinostat (HDAC inhibitor), pacritinib (JAK inhibitor), MLN2238 (proteasome inhibitor), or KPT-185 (CRM1 inhibitor). Cell death events were quantified by PI staining and flow cytometry analysis. RSL3 at 1 μM or erastin at 2 μM did not induce strong ferroptotic cell death. Treatment with other chemicals (except for MLN2238) at 1 μM did not induce massive cell death (Fig. 5A and B). However, the joint application of ferroptosis inducer and these chemicals triggered a significant increase of cell death events when compared with the ferroptosis inducer or chemical treatment alone. Notably, the application of ferroptosis inhibitor (Ferrostatin-1 [26]) largely repressed the cell death induced by the co-treatment (Fig. 5A and B). Among the chemicals, MLN2238 and ferroptosis inducer together induced the strongest effect of cell death (Fig. 5B). Besides, inhibiting proteasome activity by MLN2238 impaired the cellular capacity to antagonize H_2_O_2_-induced oxidative stress (Fig. 5C). We also validated the synergistic effect of MLN2238 and RSL3 in the 3D spheroid culture of U-2OS cells. RSL3 or MLN2238 treatment alone caused partial cell death in the outer layer of the spheroid, and their joint application induced massive cell death and the collapse of spheroids in the 3D culture (Fig. 5D). Thus, these data suggest that compounds with peroxisome-augmenting activity could potentiate the sensitivity to ferroptosis induction.

## Discussion

In this study, we established a U-2OS cell line expressing a fluorescent peroxisomal tag and screened a target-selective inhibitor library. We showed that peroxisomal abundance is closely linked with cell cycle progression and the biogenesis of cellular peroxisomes occurs mainly in the G1/S phase transition. By integrating the DNA staining signal, we identified compounds that positively and negatively regulated peroxisomal abundance without significantly affecting the cell cycle distribution. Since the protein targets of these compounds are well established, our data provide novel insights into the potential cellular targets involved in peroxisomal biogenesis.

Currently, more than 30 peroxisomal membrane proteins and peroxins have been reported in the de novo synthesis, dynamic fusion/fission, and degradation of peroxisomes [18, 27]. Defects in 14 genes encoding peroxisomal proteins are implicated in peroxisome biogenesis disorders (PBDs) [21, 28]. Peroxisomal genes related to fatty acid β-oxidation and organelle division are regulated by peroxisome proliferator-activated receptors (PPARs) [14, 29–31]. Peroxisome proliferator-activated receptor-alpha (PGC-1α) functions as a transcriptional co-activator to promote peroxisomal remodeling and biogenesis [32, 33]. In addition, the accumulation of ubiquitination in PEX5 can serve as an autophagy signal to eliminate defective and dysfunctional peroxisomes [34, 35]. Although the main players in the proliferation and degradation of peroxisomes have been characterized, little is known about the connection and coordination between these two opposing processes. Research on peroxisome homeostasis is restricted to the functional exploration of peroxisomal proteins. The dialog between different cell signals and peroxisome homeostasis is not well understood.

We demonstrated that chemicals arresting the cell cycle at the G1/S or G2/M phase promoted peroxisomal abundance, which could be attributed to the proliferation of peroxisomes in the G1 phase before entering the S phase. This is in line with the notion that active protein synthesis and organelle proliferation occur in the G1 phase [36]. In addition, inhibiting peroxisome proliferator-activated receptor PPARγ could induce cell cycle arrest at the G1/S transition [37, 38]. Our data also indicate that extra caution should be paid to exclude the confounding factor of cell cycle change when studying peroxisomal biogenesis. For example, HDAC inhibitor was reported to increase peroxisome gene expression [39]. However, these effects might be related to the cell cycle arrest induced by HDAC inhibition.

We identified inhibitors that positively or negatively regulate peroxisomal abundance without altering the cell cycle distribution. Inhibitors of proteasome, exportin 1 (CRM1), autophagy, ubiquitin-conjugating enzyme (E2) complex, JAK, and eEF2 kinase increased peroxisomal abundance, and inhibitors of ATM/ATR, adrenergic receptor, cAMP synthesis, GPR, LPA receptor, NF-kappa B signaling, and different metabolic enzymes (dehydrogenase, hydroxylase, fatty acid amide hydrolase (FAAH)) showed the opposite effects. Some of these targets have been implicated in peroxisomal quality control. For instance, ubiquitin-conjugating E2 enzyme is involved in PEX5 protein recycling during matrix protein import, and the overaccumulation of ubiquitinated PEX5 induces pexophagy [40, 41]. Proteasome-dependent degradation of peroxisomal membrane protein Pxa1p is essential for peroxisomal quality control [42]. It was also found that ATM translocates to the peroxisome under oxidative stress, triggering PEX5 phosphorylation and the subsequent pexophagy [3]. Further, the cellular metabolism state of fatty acid and lipid may impinge on peroxisomal biogenesis, since the de novo biogenesis of peroxisomes and lipid droplet is tightly coordinated [43, 44]. Although how these inhibitors modulate peroxisomal biogenesis is largely unknown, our data provide novel targets for studying peroxisomal biogenesis regulation. Future works are warranted to investigate how these protein targets regulate the protein composition, functional state, and quality of the peroxisome.

We further demonstrated that some inhibitors may promote peroxisomal abundance by inducing oxidative stress. Nevertheless, proteasome and CRM1 inhibitor increased peroxisomal abundance regardless of the ROS state. The inhibitors that reduced peroxisomal abundance seem to exert their effect in an ROS-independent manner. Whether elevated ROS level promotes peroxisomal biogenesis or favors the degradation is controversial. There is evidence that oxidative stress (such as H_2_O_2_) can enhance peroxisome biogenesis by upregulating the expression of PEX genes [8]. On the other hand, excessive oxidative damage promotes peroxisomal degradation and represses the biogenesis [45, 46]. Our data showed that inhibitors could increase peroxisomal abundance in both ROS-dependent and ROS-independent manners. The underlying mechanisms remain to be clarified.

Ferroptosis is a novel form of iron-dependent cell death caused by lipid peroxide accumulation and the loss of membrane integrity [47–49]. Inducing ferroptotic cell death has become an attractive strategy in cancer treatment, especially for patients with acquired resistance to existing therapies [50–52]. Peroxisomal activity and abundance have been recently implicated in ferroptosis susceptibility. A genome-wide CRISPRi screening study reported that multiple peroxisomal genes (Pex1, Pex2, Pex3, Pex6, Pex10, Pex12, Pex13, Pex14, Pex16, and Pex19) were enriched in the cell population surviving ferroptosis induction, and genetic deletion of Pex3, Pex10, or Pex12 conferred ferroptosis resistance by reducing peroxisomal abundance [25]. In addition, peroxisomal enzymes involved in lipid metabolism, such as acyl-CoA synthetase long-chain family member 4 (ACSL4), alkylglycerone phosphate synthase (AGPS), and fatty acyl-CoA reductase 1 (FAR1), were also identified as the top hits. Genetic ablation of FAR1 promoted the resistance to ferroptosis, which was abrogated after ectopic FAR1 expression [22]. These findings pinpoint the critical role of peroxisomal homeostasis and activity in dictating ferroptosis sensitivity. In line with this, our data showed that compounds that promoted peroxisomal abundance could enhance the susceptibility to ferroptosis induction. These compounds may be used as adjuvants to boost ferroptosis induction in cancer therapy. Nevertheless, whether these inhibitors enhance ferroptosis sensitivity by increasing peroxisomal unsaturated phospholipids synthesis or disrupting the redox balance needs further clarification.

## Conclusions

We identified different target-selective inhibitors that are capable of positively or negatively regulating peroxisomal abundance. Our findings suggest novel cellular targets for studying peroxisomal biogenesis regulation. Further, we demonstrated the pro-ferroptosis potential of compounds with peroxisome-enhancing activity. These target-selective inhibitors may be jointly applied with ferroptosis inducers to potentiate the power of anticancer effect.

## Data Availability

Upon reasonable request.

## Abbreviations

ACSL4: Acyl-CoA synthetase long-chain family member 4
AGPS: Alkylglycerone phosphate synthase
ATM: Ataxia-telangiectasia mutated
ATR: Ataxia telangiectasia and Rad3 related
CDK: Cyclin-dependent kinase
c-JUN: C-Jun N-terminal kinase
CXCR2: CXC motif chemokine receptor 2
DCFDA: 2′,7′-Dichlorodihydrofluorescein diacetate
DMSO: Dimethylsulfoxide
DPP-4: Dipeptidyl peptidase 4
eEF2: Eukaryotic elongation factor 2
FAAH: Fatty acid amide hydrolase
FAR1: Fatty acyl-CoA reductase 1 (FAR1)
Fer-1: Ferrostatin-1
GPR: G-protein coupled receptor
HDAC: Histone deacetylase
IF: Immunofluorescence
JAK: Janus kinase
LPA: Lysophosphatidic acid
MAVS: Mitochondrial antiviral signaling protein
MEK: Mitogen-activated protein kinase
NAC: *N*-acetyl cysteine
NF-kappa B: Nuclear factor-kappa B
PBDs: Peroxisome biogenesis disorders
PEXs: Peroxins
PGC-1α: Peroxisome proliferator-activated receptor-alpha
PI: Propidium iodide
PLK: Polo-like kinase
PMP70: 70-kDa peroxisomal membrane protein
PPARs: Peroxisome proliferator-activated receptors
RCDP: Rhizomelic chondrodysplasia punctata
RIPA: Radioimmunoprecipitation assay buffer
ROS: Reactive oxygen species
